# The Pediatric Ependymoma Protein Database (PEPD)

**DOI:** 10.1016/j.dib.2017.10.001

**Published:** 2017-10-17

**Authors:** George Th. Tsangaris, Athanasios K. Anagnostopoulos

**Affiliations:** Proteomics Research Unit, Biomedical Research Foundation of the Academy of Athens, Athens, Greece

**Keywords:** Pediatric brain tumors, Ependymoma, Ependymoma protein database

## Abstract

Proteomics, through application of modern high-end mass spectrometric (MS) approaches, offers the advantage of in-depth analysis of cancer tissues regarding their protein composition. Pediatric brain tumor malignancies are scarcely approached by modern holistic technologies (e.g. genomics, proteomics) due to rarity of samples and most importantly difficulty in their collection. Ependymoma, is the third most common tumor in children and is thought to arise from ependymal cells in the wall of the cerebral ventricles or the spinal canal. Due to the heterogeneity of the disease its biological characteristics remain largely unknown and prognostic factors are basically based on clinical and histological criteria. Through application of a modified nanoLC-MS/MS analysis experimental approach we achieved, for the first time, the in-depth examination of the pediatric ependymoma tissue proteome. In the present article we report on the construction of a high-confidence database; the Pediatric Ependymoma Protein Database (PEPD), including 4,157 protein groups originating from the identification and analysis of more than 15,000 peptides. The PEPD offers a first step towards targeted protein validation of the protein content of this specific devastating disease affecting the young population.

**Specifications Table**

**Table:** 

Subject area	*Proteomics, LC-MS/MS, Pediatric Brain Tumors*
More specific subject area	*Pediatric Ependymoma Proteome*
Type of data	*Excel file, Figure*
How data was acquired	1D-nanoLC-MS/MS, bottom-up proteomics
	Dionex Ultimate 3000 nanoHPLC system coupled to an LTQ Velos *Orbitrap Elite mass spectrometer* (Thermo Scientific, Rockford, IL, USA)
	PepMap® RSLC, C18, 100 Å, 3-μm-bead-packed 15-cm column and 2-μm-bead-packed 50-cm column (Thermo Scientific)
	Proteome Discoverer 1.4 software (Thermo Scientific), Sequest search engine searching the *Homo Sapiens*.fasta database
Data format	*Analyzed*
Experimental factors	*Resected specimens of ependymoma patients*
Experimental features	*Whole-tissue proteome analysis*
Data source location	*Athens, Greece*
Data accessibility	*Dataset is directly provided with this article*

**Value of the data**

• An enriched proteome dataset of pediatric ependymoma proteins is reported for the first time.
• The data can be used to govern future steps towards targeted protein validation of the protein content of this devastating disease affecting the young
• Major components of the PI3K pathway, seemingly implicated in ependymoma pathogenesis are included in the database
• The majority of ependymal tissue proteome (> 60%) is presented in the present database
• More than 5,000 proteins entries, belonging to 4,157 protein groups and 15,675 peptides are included in the final PEPD

## 1 Data

**Fig. 1:**
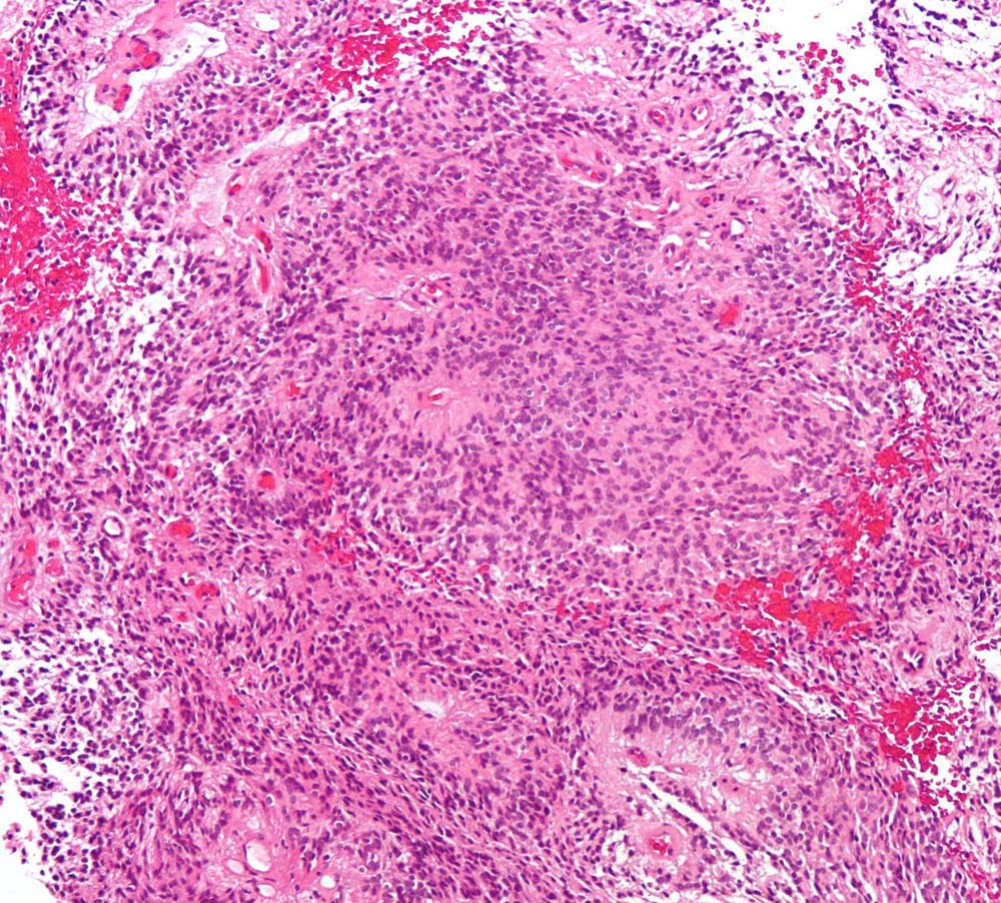
Microphotograph of an ependymoma tissue. An H&E-stained section is shown.

In the present study we aimed on an in-depth analysis of the protein content of human pediatric ependymoma tumor resections. Ten resection specimens, after immunohistochemical characterization and H&E staining (Fig. 1), were subjected to 1D-nanoLC-MS/MS bottom-up proteomic analysis. To further strengthen the quality and reproducibility of protein profiling for each tissue sample, our approach consisted of two technical replicates of each section. The undertaken methodology, aiming at achieving the highest possible identification rate, consisted of a modified experimental protocol with regard to the protein extraction procedure as well as improved parameters in liquid chromatography.

The final PEPD consists of total of 4,157 proteins (protein groups) that were identified in common in all ten tumor samples analyzed (Table 1). In order for proteins to be included in the final PEPD, they had to be present in at least 9 out of 10 analyzed samples. Table 1 lists all identified pediatric ependymoma proteins, also showing their accession number, peptides identified in each protein, and their description according to the Uniprot database.

## 2 Experimental design, materials and methods

### 2.1 Samples and patient characteristics

All specimen analyzed in the study belonged to WHO grade II ependymomas and all individuals were Caucasian. Immediately after tumor resection tissues were analyzed for protein extraction without interference of any freeze-thaw cycles.

### 2.2 Sample preparation

Prior to experiments, all tissue samples were washed in a sucrose buffer (pH 7.5, 320 mM sucrose) for washing-off remaining blood. Tissue lysis was performed in liquid N_2_ and next samples were homogenized with tip sonication in lysis buffer (7 M Urea, 1.5 M Tris–HCl, 0.1 M SDS). The protein content of whole-tissue lisates was estimated with the Bradford assay [1]. Finally 150 μg of protein were further processed for peptide generation.

### 2.3 Peptide generation and 1-D nanoLC-MS/MS analysis

Protein extraction and peptide generation, was done as described by our group elsewhere, with few modifications [2]. In brief, reduction and alkylation steps of protein solutions were carried-out using dithiothreitol and iodoacetamide solutions, at concentrations of 10 mM and 55 mM, respectively. The final step included tryptic digestion of extracted proteins for generation of peptide. Trypsin (Roche Diagnostics, Basel Swiss) at a final concentration of 500 ng/μl, was applied to all samples in a humidified atmosphere, and samples were left to digest overnight.

### 2.4 NanoLC-MS/MS analysis

Digested samples were analyzed using a LTQ Orbitrap Elite coupled to a Dionex 3000 nanoHPLC system (Thermo Scientific, Rockford, IL, USA), as described previously [3]. LC separation of peptides took place on two Thermo Scientific columns (PepMap® RSLC, C18, 100 Å, 3-μm-bead-packed 15-cm column and 2-μm-bead-packed 50-cm column) at a flow rate of 300 nL/min. The mobile phases A and B were 0.1% formic acid in water and 99.9% ACN in H_2_O, respectively. Data were collected in the data-dependent MS/MS mode using a standard top-20 method. Full-scan data were acquired at a resolving power of 60,000 with a maximum integration time of 250 msec. Scan range was fixed at 250 to 1,250 m/z and peptide fragmentation was performed in a higher-energy collision dissociation (HCD) mode with a normalized collision energy of 36%. MS/MS spectra were acquired with 15,000 resolving power and a maximum integration time of 120 ms. Measurements were performed using m/z 445.1 as lock mass.

The.raw data files were analyzed using the Proteome Discoverer software (Thermo Scientific), using the Sequest search engine applying the *Homo Sapiens*.fasta databases. MS/MS searches were performed using a 10 ppm parent ion mass tolerance and a 0.05 fragment mass tolerance. Trypsin was selected as the cleavage enzyme with up to 2 missed cleavage points. Cysteine methylthio modification was selected as a fixed modification and oxidation of methionine were selected as a variable. Peptide identifications were considered valid at 1% False Discovery Rate (q-value < 0.01) (percolator maximum Delta Cn was 0.05). The minimum length of acceptable identified peptides was set as 6 amino acids.

## Appendix A Supplementary material

Supplementary material

Supplementary material Protein groups included in the PEDB have been identified in at least 9 out of 10 analyzed samples.
