# Supplementary material for: The Pediatric Ependymoma Protein Database (PEPD)
Source: Data Brief. 2017 Oct 17;15:638–41. doi: 10.1016/j.dib.2017.10.001 (PMC5671472; doi:10.1016/j.dib.2017.10.001)
Supplement: Supplementary file 1 — Transparency document [file mmc1.docx]

**Conflict of Interest Form**

The Authors declare there exist no conflicts of interest with regards to this study.
